# Amebiasis as a neglected tropical disease: Current knowledge gaps and future directions

**DOI:** 10.1371/journal.pntd.0014423

**Published:** 2026-06-22

**Authors:** Debbie-Ann Shirley, Shasika Jayarathne, Cirle A. Warren, Shannon Moonah

**Affiliations:** 1 Department of Pediatrics, Division of Infectious Diseases, University of Florida College of Medicine, Gainesville, Florida, United States of America; 2 Department of Medicine, Division of Infectious Diseases, University of Florida College of Medicine, Gainesville, Florida, United States of America; 3 Department of Medicine, Division of Infectious Diseases and International Health, University of Virginia, Charlottesville, Virginia, United States of America; 4 Department of Molecular Genetics & Microbiology, University of Florida College of Medicine, GainesvilleFlorida, United States of America; Institute of Continuing Medical Education of Ioannina, GREECE

## Abstract

*Entamoeba histolytica* is a protozoan parasite that causes amebic colitis, a leading cause of severe diarrheal disease worldwide, and amebic liver abscess, the most common extraintestinal manifestation of infection. The disease burden is highest in resource-limited settings but remains clinically important in travelers and men who have sex with men in non-endemic regions. Although most infections are asymptomatic, severe and fulminant disease is associated with high mortality, particularly among individuals exposed to corticosteroids or other forms of immunosuppression. The increasing use of molecular diagnostic tools has improved understanding of the epidemiology of *E. histolytica* and enabled distinction from morphologically identical but nonpathogenic *Entamoeba* species; however, these diagnostics remain underutilized in many endemic settings due to cost and infrastructure limitations. Treatment options remain limited, with nitroimidazoles constituting the only drug class available for symptomatic invasive disease, leaving few alternatives for patients who cannot tolerate therapy or in the event of emerging resistance. Despite advances in understanding parasite pathogenesis and the application of high-throughput technologies, no licensed vaccine exists, and progress toward vaccine development has been minimal. These persistent gaps highlight the need to reprioritize amebiasis as a neglected tropical disease and to accelerate investment in diagnostics, therapeutics, and preventive strategies.

## Introduction

Amebiasis is caused by infection with the protozoan parasite, *Entamoeba histolytica* [1]. Infection can be asymptomatic or progress to invasive disease, including amebic colitis and amebic liver abscess. Despite its long-standing global impact, amebiasis remains a significantly neglected parasitic disease. Amebic colitis is a leading cause of severe diarrhea and ranks among the top 15 causes of diarrheal illness in children during the first two years of life in low-resource settings, where diarrhea remains among the top five leading causes of childhood mortality [2,3]. Fulminant forms of amebiasis are rare but are associated with remarkably high mortality [4].

**Fig 1:**
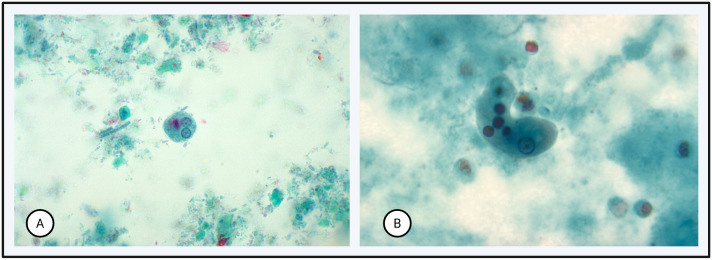
Life Stages of *Entamoeba histolytica.* **A)** Trichome stain of immature binucleated cyst stage, mature cysts have 4 nuclei measuring about 12–15 µm in diameter. **B)** Trichome stain of trophozoite, which has a single nucleus and is slightly larger than the cyst stage, measuring about 15–20 µm. A number of ingested red blood cells are also seen (erythrophagocytosis). Image courtesy of the Public Health Image Library (PHIL), CDC.

The parasite’s life cycle, which alternates between the environmentally resistant infectious cyst and the tissue-invasive trophozoite (Fig 1), facilitates efficient fecal-oral transmission, particularly in environments lacking access to adequate sanitation and hygiene [1,5,6]. Consequently, the highest disease burden occurs in resource-limited settings. In non-endemic settings, infection is typically travel-associated or sexually transmitted [7–10]. *E. histolytica* infection remains an important differential diagnosis for persistent diarrhea, inflammatory bowel disease mimics, and pyogenic liver abscess.

The advent of molecular diagnostics has enabled reliable differentiation of *E. histolytica* from morphologically identical non-pathogenic species. However, the burden of amebiasis, including the impact of asymptomatic infection to child health, remains poorly understood, and several regions of the world continue to report high prevalences. Although these state-of-the-art tools also facilitate accurate diagnosis, their availability and use remain limited globally, particularly in endemic settings.

Despite advances in understanding parasite adhesion, invasion, phagocytosis, and interactions with the host immune system and microbiota, progress made in treatment and prevention has been limited. Nitroimidazoles remain the only drug class available for treatment of the tissue-invasive disease, with no approved alternatives for patients who cannot tolerate therapy, and no licensed vaccine. Few new therapies are in development, reflecting sustained underinvestment in a disease that disproportionately affects vulnerable populations. Amebiasis therefore represents a paradigmatic neglected tropical enteric infection, characterized by substantial disease burden, diagnostic limitations, and stagnant therapeutic progress.

This review summarizes recent advances in epidemiology, clinical manifestations, laboratory diagnosis, and management of amebiasis, with particular emphasis on developments over the past five years and ongoing areas of unmet need. A structured PubMed search was conducted on December 8, 2025, identifying 377 English-language articles published within the preceding five years, which were independently screened by title and abstract for relevance, incorporating those most pertinent to this review.

## Epidemiology

Advances in molecular diagnostics, including PCR and sequencing, are increasingly supplanting traditional microscopy, enabling accurate differentiation of pathogenic *E. histolytica* from morphologically identical, nonpathogenic species, namely *E. dispar*, *E. moshkovii*, and *E. bangladeshii* [11]. Despite these advances, the current global epidemiology of infection remains poorly understood. While the incidence appears to be declining in some regions, a substantial burden persists in other low-resource and marginalized populations across Africa, Asia, Central, and South America [5].

In the large Global Enteric Multi-Center Study (GEMS), *E. histolytica* ranked among the top 10 causes of moderate-to-severe diarrhea in children under five at two of seven study sites across sub-Saharan Africa and South Asia [12]. Conversely, in the multisite birth cohort (MAL-ED) study, *E. histolytica* was infrequently detected and not strongly associated with diarrhea in early childhood, yet recent antigen-based and molecular surveillance studies continue to document significant prevalence among school-aged children and vulnerable populations in many areas [13]. For instance, a school-based cross-sectional study in Dilla, Ethiopia, reported 13.2% positivity by antigen detection [14], while PCR-based screening in Perak, Malaysia, identified *E. histolytica* in the stool of 5.0% of school children [15]. In Manila, Philippines, PCR detected *E. histolytica* in 16.4% of residents in an urban slum [16], compared with 0.8% prevalence by antigen detection in a separate community survey in BASECO [17]. These discrepancies highlight persistent but variable endemicity and illustrate how methodological differences influence prevalence estimates. Additional studies report 7.5% positivity by PCR among 895 patients with diarrhea or abdominal discomfort in the Mukuru settlement in Nairobi, Kenya [18], and approximately 5% positivity among patients presenting with gastrointestinal symptoms in northern and southern India [19,20]. National surveillance data from Mexico demonstrate a relatively stable incidence of 0.47 cases per 100,000 population annually for amebic liver abscess over a 7-year period [21].

Although global mortality from diarrheal diseases is declining, the absolute burden remains substantial. In 2023, enteric infections caused 1.27 million deaths worldwide across, disproportionately affecting children and the elderly, particularly in sub-Saharan Africa and South Asia [22]. Amebic colitis has historically been estimated to cause over 55,000 deaths annually representing a leading parasitic cause of death [23]. Fulminant amebiasis, while rare, carries a case fatality rate exceeding 20% [4,24,25].

In high-income countries, such as the United States, amebiasis is uncommon but is the third most frequently identified pathogen in returning travelers seeking care for gastrointestinal symptoms [7]. Sexual transmission is increasingly recognized, particularly among men who have sex with men in Asia, Europe and North America [9,15,26]. Endemic transmission may occur in some underserved communities, as evidenced by soil contamination, though contemporary data are limited because amebiasis is no longer reportable in many jurisdictions [27].

Transmission occurs primarily via the fecal–oral route following ingestion of environmentally resilient cysts that contaminate hands, food, or water, with sexual transmission also documented [5,6,9,15,26]. Humans are the only known reservoir. After excystation in the small intestine, trophozoites migrate to the colon, where infection may remain asymptomatic or progress to invasive intestinal or extraintestinal disease. The cyst’s resistance to gastric acid, chlorination, and environmental stressors combined with a low infectious dose, facilitates persistent, often silent transmission in resource-limited settings [28]. Clinical manifestations may develop within weeks after initial infection, but can also develop years later, complicating surveillance and burden estimation [29]. These features underscore the need for integrated interventions combining sanitation, hygiene, and improved surveillance.

## Pathogenesis

The wide range of clinical outcomes observed in amebiasis reflects complex interactions among parasite virulence, host immune responses, and environmental factors. Genetic diversity among *E. histolytica* isolates further contributes to variability in disease severity.

Pathogenesis can be broadly conceptualized as involving host cell death, inflammation, and tissue invasion. Infection begins with parasite adhesion to intestinal epithelial cells through surface adhesins, such as the Gal/GalNAc lectin. Adhesion to host cells is a critical determinant of *E. histolytica*–induced cell death. Host cell damage is mediated through multiple mechanisms, including induction of programmed cell death, phagocytosis, and trogocytosis. Subsequent secretion of parasite cysteine proteases, along with induction of host matrix metalloproteinases (MMPs), leads to degradation of the extracellular matrix and protective mucus layer, collectively enabling mucosal invasion. The parasite-derived macrophage migration inhibitory factor homolog (MIF) further amplifies inflammation by inducing host cytokine responses and upregulating MMP expression [30,31]. These processes are further shaped by immune evasion strategies and resistance to oxidative stress [30,31]. Host susceptibility is also influenced by microbiome composition and clinical factors such as age, pregnancy, immunosuppression, and alcohol use [32–35]. The complex pathogenesis of Entamoeba histolytica infection has been well-reviewed elsewhere [30,36]

## Clinical considerations

Most individuals infected with *E. histolytica* remain asymptomatic, with only an estimated 10%–20% developing clinically apparent disease. Symptoms arise following trophozoite invasion of the colonic mucosa resulting in tissue destruction, ulceration, and inflammation. Endoscopic studies have identified asymptomatic patients with invasive disease, underscoring the complexity of host-parasite interactions [33]. Progression to symptomatic infection likely reflects variation in parasite virulence, host immune regulation, nutrition, microbiome composition, and environmental exposures [32,37].

### Amebic colitis

Clinical manifestations of amebic colitis range from mild diarrhea to severe dysentery, with common associated symptoms including abdominal pain, cramping, weight loss, and malaise [10]. In non-endemic settings, careful assessment of travel, immigration, and sexual health history is critical for diagnosis [9,38,39].

**Fig 2:**
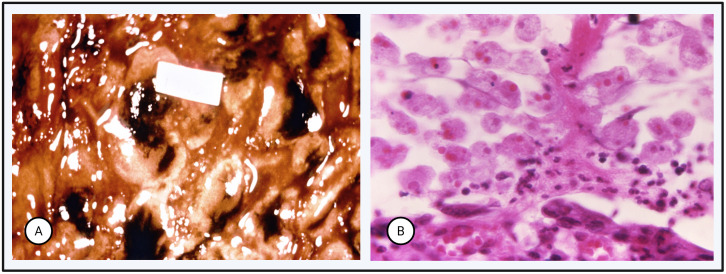
Pathologic findings of amebic colitis. **A)** Gross pathologic changes in the intestinal mucosa with multiple ulcerations. **B)** Histopathologic changes under magnification of 500× showing numerous *E. histolytica* trophozoites, some contain ingested red blood cells (erythrophagocytosis). Image courtesy of the Public Health Image Library (PHIL), CDC.

Endoscopy, when performed, may reveal friable mucosa with discrete ulcerations most commonly involving the cecum and rectum, while intervening mucosa appears normal [40]. Histopathology (Fig 2) may demonstrate trophozoites within the inflammatory exudate and superficial mucosa [40]. Characteristic flask-shaped ulcers reflect submucosal necrosis, a hallmark finding of amebiasis.

The clinical presentation of amebic colitis can closely resemble inflammatory bowel disease, creating diagnostic challenges with significant therapeutic implications, as corticosteroid exposure can precipitate severe amebic disease [40,41]. Chronic infection may result in ameboma formation, a localized inflammatory mass that can mimic malignancy [42].

Fulminant necrotizing amebic colitis is rare but can be lethal, leading to complications such as bowel necrosis, perforation, peritonitis, toxic megacolon and shock [4]. Corticosteroid exposure is a major risk factor for severe disease [4]. The association was highlighted during the COVID-19 pandemic, when multiple cases of severe amebic colitis were reported following dexamethasone exposure for the management of COVID-19 lower airway disease with hypoxemia [43–46].

### Amebic liver disease

Amebic liver abscess is the most common extraintestinal manifestation and results from hematogenous dissemination via the portal hepatic system. Amebic liver abscesses occur disproportionately in males, often 25–44 years of age and older [21,39]. The reason for male predilection is unclear, but endogenous male hormones are purported to contribute [47]. Similarly, for reasons not well understood, alcohol use is more commonly observed [47,48]. Indigenous alcoholic beverages have been proposed as a source of exposure, though this may be incidental and confounded by poor sanitation [21,49]. Alcohol related liver injury has also been suggested, but a definitive mechanism remains unestablished [47]. Clinical presentation can occur years after initial infection [29,47]. Patients typically present with acute or subacute right upper quadrant pain and fever [39]. Concurrent gastrointestinal symptoms may be absent. Physical examination may reveal hepatomegaly with focal tenderness [39]. Respiratory symptoms such as cough or pleuritic pain may occur when there is adjacent pleural involvement.

**Fig 3:**
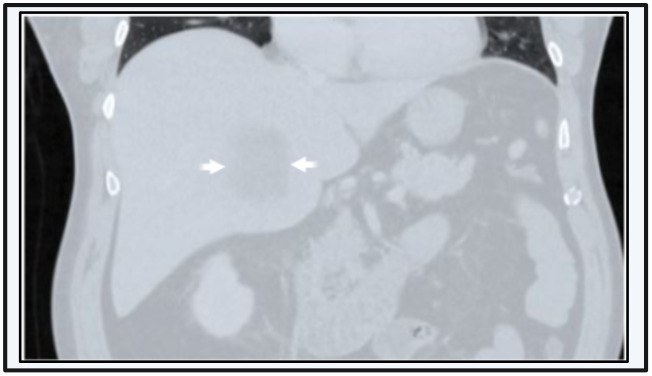
Amebic liver abscess shown on abdominal computed tomography. Returning traveler from an endemic area presenting with an amebic liver abscess seen on computed tomography (CT) imaging (white arrows).

**Fig 4:**
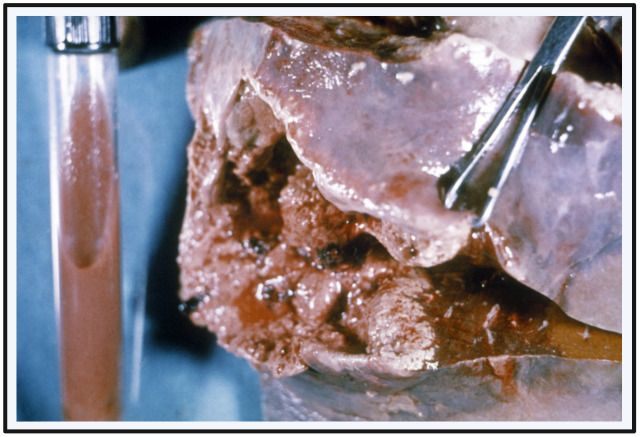
Amebic liver abscess gross pathologic features. Amebic liver abscess from a live specimen and dark brown purulent fluid extracted from the abscess. Image courtesy of the Public Health Image Library (PHIL), CDC. This image is in the public domain and thus free of any copyright restrictions.

Associated laboratory findings frequently include elevated alkaline phosphatase and peripheral leukocytosis with neutrophilic predominance. Imaging studies play a central role in diagnosis. On ultrasound, lesions typically appear as hypoechoic round masses or hypodense lesions with smooth, thin walls on computed tomography (Fig 3). Magnetic resonance imaging demonstrates peripheral rim enhancement with a non-enhancing necrotic core on post-contrast sequences. Most amebic liver abscesses are solitary and located in the right hepatic lobe, though left lobe involvement or multiple lesions may occur. Abscess rupture can lead to peritonitis or extension into adjacent structures, including the gall bladder, pleura, and pericardium [50–52]. Aspirated fluid (Fig 4) is classically brown and resembles “anchovy paste” [53]. Clinically, amebic liver abscesses can be difficult to distinguish from pyogenic liver abscesses. and laboratory testing is often required [47]. Hydatid cysts due to echinococcosis are another consideration, though these often remain asymptomatic until substantial enlargement, serologic or imaging-based evaluation can aid in differentiation [54].

Hematogenous dissemination to distant sites is also known to occur, including involvement of the central nervous system [55]. Cutaneous manifestations of extraintestinal amebiasis, although rare, have also been described [56].

## Laboratory diagnosis

Advances in antigen-based and molecular diagnostic techniques have substantially improved the detection of *E. histolytica*. PCR-based assays demonstrate superior sensitivity and specificity compared with conventional methods although diagnosis often requires a combination of approaches depending on clinical presentation and resource availability [1,20] (Table 1).

**Table 1:** Performance of laboratory tests used in the diagnosis of amebiasis.

Test	Sensitivity	Specificity	Notes
**Microscopy**	<60%	Poor/variable	Widely available and low cost, but limited by poor sensitivity and specificity. Cannot reliably distinguish *E. histolytica* from nonpathogenic *Entamoeba* species. Requires multiple stool samples and a skilled observer; time-consuming.
**Serology**	65–90%	>90%	Useful adjunct to stool testing, particularly for suspected amebic liver abscess and in travelers from nonendemic regions. Antibodies may persist for years, limiting utility for distinguishing active from prior infection in endemic settings.
**Stool Antigen Detection**	0–90%	>80%	Simple and rapid with improved performance over microscopy in some endemic settings. Sensitivity is reduced in nonendemic areas and is poor for liver abscess. Requires fresh stool samples.
**PCR**	90–100%	90–100%	Gold standard diagnostic test with excellent sensitivity and specificity for both colitis and liver abscess. Available in multiplex panels. Higher cost and need for specialized equipment and trained personnel may limit use in resource-limited settings.

Diagnostic methods for amebiasis includes dual microscopy, antigen detection, molecular testing, and serologic assays. In many resource-limited settings diagnosis continues to rely on microscopic identification of cysts and trophozoites in fresh or preserved stool. While often widely available, microscopy is time-consuming and lacks specificity because pathogenic *E. histolytica* cannot be reliably distinguished from morphologically identical nonpathogenic species [20]. In addition, the sensitivity of stool microscopy is dependent upon operator expertise, the need for intact structural features, and strict requirements for specimen handling and preservation as trophozoites may disintegrate within one to two hours after collection, leading to substandard sensitivity [20,40]. Notably, stool microscopy can be negative in patients with amebic liver abscess and other extraintestinal manifestations [39].

Stool antigen detection assays using fresh stool, including several commercially available ELISA-based tests, generally perform well in endemic settings but may show reduced sensitivity in non-endemic populations [20,57,58]. Some kits can detect other enteroparasites, however, kits are still not widely available in resource -imited areas and test performance may also be adversely affected by sample freezing or preservation methods as well as by prior antiparasitic therapy which reduces detectable antigen levels [20,59].

Molecular diagnostics including conventional and real-time PCR assays represent the most sensitive and specific methods for detecting *E. histolytica* in the stool and allow definitive species-level identification [20,57]. It is the preferred method for diagnosis when available, but cost presents a barrier in endemic settings. Multiplex PCR platforms further enable simultaneous differentiation from other enteric pathogens [60]. Despite these advantages PCR testing requires specialized equipment and technical expertise, limiting its availability in many high-burden resource-constrained settings. Use of real-time PCR and fluorescence in situ hybridization (FISH) has also been reported as a feasible diagnostic approach for *E. histolytica* using formalin-fixed, paraffin-embedded tissue submitted for histopathologic examination, although data remain limited [38]. PCR and antigen-based assays may also be applied to liver aspirate specimens to support the diagnosis of amebic liver abscess [53].

Serologic testing can be particularly useful for the diagnosis of extraintestinal amebiasis including liver abscess especially when stool studies are negative [39]. Antibodies may be absent early in infection. Although serology may be negative early in the course of infection, the persistence for years thereafter limits the ability of serologic assays to differentiate prior exposure from active disease, particularly in endemic settings [1]. Expanding access to affordable, diagnostic tools with high predictive value will be essential to improving clinical management of amebiasis as a neglected tropical infection.

## Therapy

All individuals diagnosed with *E. histolytica* infection, whether with symptoms or not, require treatment (Table 2). Symptomatic invasive disease, including amebic colitis and amebic liver abscess, require combination therapy consisting of a tissue-active amebicidal agent followed by an intraluminal cysticidal agent to eliminate residual intestinal carriage. In contrast, asymptomatic infection can be treated with a single intraluminal agent to prevent progression to invasive disease and to decrease ongoing transmission [1,61].

**Table 2:** Antiparasitic treatment dosages for *Entamoeba histolytica* infection [76,77].

	Drug of Choice	Daily Dose Adult	Daily Dose Pediatric	Duration, days	Alternative
*Tissue Active amoebicidal agent*					
Mild to moderate amebic colitis	Metronidazole, Or	500–750 mg PO TID	35–50 mg/kg/d divided TID (max 500–750 mg/dose)	7–10	Nitazoxanide^2^
	Tinidazole	2 g PO once daily	Age >3 years: 50 mg/kg once daily (max 2 g/dose)	3	
Severe amebic colitis and amebic liver disease^1^^,^^*^	Metronidazole, Or	500–750 mg IV TID	35–50 mg/kg/d IV divided TID (max 500–750 mg/dose)	7–10	Nitazoxanide^2^
	Tinidazole	2 g po once daily	Age > 3 years: 50 mg/kg once daily (max 2 g/dose)	5	
*Luminal cysticidal agent*					
Asymptomatic carriage or following tissue-active agent	Paromomycin	25–35 mg/kg/d by mouth divided TID		7	Iodoquinol^3^

BID, twice daily; IV, intravenous; PO, by mouth; TID, three times daily.

^1^Switch to oral administration when able to tolerate.

*Optimal treatment of other forms of extraintestinal amebiasis are not well defined, management is similar to severe colitis and liver abscess, with longer courses and source control as needed, guided by clinical response.

^2^Based on limited data, use age-based dosing for Nitazoxanide, not FDA approved. For ≥12 years: 500 mg po BID x 3 days; age 4–11 years: 200 mg BID x 3 days; age 1–3 years: 100 mg BID for 3 days. Take with food. Liquid formulation no longer available in the United States.

^3^Iodoquinol dose 650 mg po TID (30–40 mg/kg/d po divided TID for children) for 20 days after meals, use with caution as optic neuritis and peripheral neuropathy have been reported; not available in the United States.

At present, nitroimidazoles are the only available tissue-active agents, with metronidazole and tinidazole most commonly used. Metronidazole remains the standard of care as it is widely available but is frequently associated with adverse effects, including nausea, headache, metallic taste, anorexia, neuropathy with prolonged use, and a disulfiram-like reaction with alcohol consumption. Tinidazole offers similar efficacy with a shorter treatment course and may be better tolerated in some patients.

Surgical intervention may be necessary in cases of fulminant amebic colitis complicated by perforation, necrosis, or toxic megacolon. While amebic liver abscesses are often treated medically, percutaneous or surgical drainage may sometimes be considered for large (>5–10 cm), refractory, or complicated amebic liver abscesses [39,62].

Following completion of nitroimidazole therapy, an intraluminal agent is required to eradicate cyst carriage [47]. Paromomycin, a nonabsorbable aminoglycoside, is the preferred luminal agent in the United States. Whether all asymptomatic patients with mucosal lesions are cured is yet to be determined [32,63]. Alternative intraluminal therapies, including diloxanide furoate and iodoquinol, are used in some settings but are not available in the United States, and their utility is limited by availability and adverse effect profiles.

Aside from nitroimidazoles and intraluminal agents, therapeutic options for amebiasis remain extremely limited, underscoring the need for safe, effective, and accessible alternatives (Table 3). Nitazoxanide has been evaluated as an alternative therapy for amebiasis. A randomized trial conducted in the Nile delta of Egypt reported that nitazoxanide was highly effective for treating intestinal amebiasis, achieving a 94% microbiologic cure versus 43% with placebo, but the trial was limited by a small sample size, short follow-up, single geographic setting, and high placebo response [64]. A second trial conducted in Jaipur, India, found that nitazoxanide was as effective as metronidazole for treating uncomplicated amebic liver abscess, with similar clinical and radiologic cure rates and fewer adverse effects, but the study was also limited by a modest sample size, single-center design, with follow-up limited to 6 months [65]. Recurrence of amebic liver abscess can be as high as 9% within 2 years of treatment [48].

**Table 3:** Anti-parasitic drugs for amebic colitis including approved treatments and agents under clinical development.

Drug (Class)	Mechanism/Target	Clinical Status/Use	Notes/Limitations
*Tissue active agents* ^*^			
**Metronidazole** (Nitroimidazole)	DNA damage	FDA-approved, first-line	Highly effective, but side effects can make difficult to tolerate treatment course; potential emerging reduced susceptibility
**Tinidazole** (Nitroimidazole)	DNA damage	FDA-approved	Similar efficacy to metronidazole; shorter course and better tolerability in some patients
**Ornidazole and Secnidazole** (Nitroimidazole)	DNA damage	Approved in some countries	Evidence limited; similar mechanism as metronidazole
*Luminal*			
**Paromomycin** (Aminoglycoside)	Protein synthesis inhibition	FDA-approved, first line	Clears cysts in intestine; not absorbed; used after tissue-active therapy or asymptomatic carriage
**Iodoquinol** (Halogenated hydroxyquinoline)	Disrupts metal-dependent enzymatic processes	Used in some countries	Clears intestinal cysts, associated with optic neuritis particularly with prolonged use and availability varies widely by country (not available in the US)
**Diloxanide furoate** (Dichloroacetamide)	Disrupts parasite metabolism	Used in some countries	Clears intestinal cysts; availability limited (not available in the US)
*Under clinical phase development*			
**Nitazoxanide** (Thiazolide)	Inhibits pyruvate:ferredoxin oxidoreductase	Not FDA-approved	Limited data for use in non-severe amebic colitis and amebic liver abscess
**Auranofin** (Gold)	Inhibits parasite redox enzymes	Investigational; Phase I, Phase IIa completed	Under investigations as a repurposed drug, limited enrollment may impact safety and efficacy outcomes
**Disulfiram + Zinc** (Aldehyde dehydrogenase inhibitor)	Novel combination, inhibition of parasite protein degradation	Early-stage clinical development	Not yet recruiting; repurposed indication of an inexpensive, widely available alcohol abuse drug with established PK and safety data

*Historical use of chloroquine and dehydroemetine, but inferior efficacy and toxicity limits modern use; *FDA*, US Food and Drug Administration.

Beyond these agents, therapeutic options for amebiasis remain extremely limited for patients who cannot tolerate nitroimidazole therapy or if metronidazole resistance emerges. Resistant strains are readily generated in the laboratory, and recent data indicate that inhibitory concentrations (IC₅₀) of nitroimidazoles against clinical *E. histolytica* isolates may be rising in some areas relative to historical reference values, potentially portending gradual susceptibility loss [66]. In this context, drug repurposing represents a pragmatic and attractive strategy, offering faster clinical translation at lower cost by leveraging compounds with established pharmacokinetic properties and human safety data. Auranofin, a repurposed gold compound, has completed a Phase IIa clinical trial (NCT02736968); however, the enrollment of only a single participant with confirmed amebiasis will substantially limit interpretation of its efficacy and safety [67]. A randomized clinical trial evaluating the combination of the repurposed, inexpensive, and widely available alcohol abuse drug, disulfiram combined with zinc supplement is currently underway (ISRCTN15356736) [68–70].

## Prevention

Despite the substantial global burden of amebiasis, no licensed human vaccine is currently available. Prevention relies on improved sanitation, clean water access, and hygiene. Vaccine development has primarily remained in the preclinical stage, with efforts focused on parasite antigens critical to host invasion and pathogenicity. The most extensively studied candidate is the Gal/GalNAc lectin, given the key role played in adhesion [71]. Vaccines incorporating recombinant or synthetic peptides derived from the lectin heavy chain have demonstrated partial protection in animal models when administered with appropriate adjuvants [71]. Preclinical studies have evaluated intranasal delivery of LecA-based vaccines formulated with GLA-3M-052 adjuvant in liposomes, demonstrating reproducible spray deposition and minimal lung penetration in adult and infant airway models, supporting the feasibility of mucosal vaccination strategies for amebiasis [72]. Another investigational target, the serine-rich *Entamoeba histolytica* protein (SREHP), has shown reduced liver abscess formation in gerbil and murine models using DNA-based vaccine platforms [73]. More recently, computational approaches have enabled the design of multi-epitope vaccine candidates targeting surface proteins expressed in both trophozoite and cyst stages [74]. However, none of these candidates have progressed to human clinical trials, and ongoing challenges to vaccine development include incomplete understanding of protective immune correlates, antigenic diversity, and limited commercial investment [75].

## Conclusion

Notwithstanding recent advances in molecular diagnostics and fundamental research, amebiasis remains a neglected tropical disease with substantial global burden, limited therapeutic options, and no licensed vaccine. Addressing persistent knowledge gaps in epidemiology, expanding access to accurate diagnostics, and accelerating the development of new treatments and preventative strategies are critical priorities.

**Key learning points**

- Amebiasis remains a major cause of severe diarrheal disease and liver abscess worldwide, with the highest burden in resource-limited settings and continued clinical relevance in travelers as well as men who have sex with men in non-endemic regions.
- Accurate diagnosis of the causative parasite, *Entamoeba histolytica*, requires molecular or antigen-based testing, as microscopy cannot reliably distinguish pathogenic from nonpathogenic Entamoeba species, however, access to these diagnostics remains limited in many endemic areas.
- Therapeutic options for invasive amebiasis are narrowly restricted to nitroimidazoles, leaving limited alternatives for patients who cannot tolerate therapy and raises concern about preparedness should drug resistance emerge.
- Despite advances in understanding parasite biology and host-pathogen interactions, no licensed vaccine exists, underscoring the need for renewed investment in prevention, drug development and equitable access to diagnostics.

**Five key papers**

1. Yanagawa Y, Shimogawara R, Takano M, Aoki T, Mizushima D, Gatanaga H, et al. Identification of asymptomatic Entamoeba histolytica infection by a serological screening test: A cross-sectional study of an HIV-negative men who have sex with men cohort in Japan. PLoS Negl Trop Dis. 2022;16(4):e000979
2. Guillén N. Pathogenicity and virulence of *Entamoeba histolytica*, the agent of amoebiasis. Virulence. 2023 Dec;14(1):2158656. https://doi.org/10.1080/21505594.2022.2158656. PMID: 36519347; PMCID: PMC9815260.
3. Gonzales MLM, Dans LF, Sio‐Aguilar J. Antiamoebic drugs for treating amoebic colitis. Cochrane Database of Systematic Reviews. 2019(1). PMID: 30624763; PMCID: PMC6326239.
4. Singh A, Banerjee T, Shukla SK, Upadhyay S, Verma A. Creep in nitroimidazole inhibitory concentration among the Entamoeba histolytica isolates causing amoebic liver abscess and screening of andrographolide as a repurposing drug. Sci Rep. 2023 Jul 27;13(1):12192. https://doi.org/10.1038/s41598-023-39382-1. PMID: 37500681; PMCID: PMC10374660.
5. Shrivastav MT, Malik Z, Somlata. Revisiting Drug Development Against the Neglected Tropical Disease, Amebiasis. Front Cell Infect Microbiol. 2021 Feb 24;10:628257. https://doi.org/10.3389/fcimb.2020.628257. PMID: 33718258; PMCID: PMC7943716.
